# A Real-Time Recognition System of Driving Propensity Based on AutoNavi Navigation Data

**DOI:** 10.3390/s22134883

**Published:** 2022-06-28

**Authors:** Xiaoyuan Wang, Longfei Chen, Huili Shi, Junyan Han, Gang Wang, Quanzheng Wang, Fusheng Zhong, Hao Li

**Affiliations:** 1 College of Electromechanical Engineering, Qingdao University of Science & Technology, Qingdao 266000, China; chenlongfei@mails.qust.edu.cn (L.C.); shihuili@qust.edu.cn (H.S.); hanjunyan@mails.qust.edu.cn (J.H.); wanggang@mails.qust.edu.cn (G.W.); 0020030005@mails.qust.edu.cn (Q.W.); bh136@qust.edu.cn (F.Z.); 4019030012@mails.qust.edu.cn (H.L.); 2 Collaborative Innovation Center for Intelligent Green Manufacturing Technology and Equipment of Shandong, Qingdao 266000, China

**Keywords:** drivers, driving propensity, intelligent driving assistant system, AutoNavi navigation data

## Abstract

Driving propensity is the driver’s attitude towards the actual traffic situation and the corresponding decision-making or behavior during the driving process. It is of great significance to improve the accuracy of safety early warning and reduce traffic accidents. In this paper, a real-time identification system of driving propensity based on AutoNavi navigation data is proposed. The main work includes: (1) A dynamic data acquisition method of AutoNavi navigation is proposed to obtain the time, speed and acceleration of the driver during the navigation process. (2) The dynamic data collection method of AutoNavi navigation is analyzed and verified through the dynamic data obtained in the real vehicle experiment. The principal component analysis method is used to process the experimental data to extract the driving propensity characteristics variables. (3) The fruit fly optimization algorithm combined with GRNN (generalized neural network) and the feature variable set are used to build a FOA-GRNN-based model. The results show that the overall accuracy of the model can reach 94.17%. (4) A driving propensity identification system is constructed. The system has been verified through real vehicle test experiments. This paper provides a novel and convenient method for building personalized intelligent driver assistance systems in practical applications.

## 1. Introduction

Human factors account for more than 90% of traffic accidents. More than 70% of traffic accidents are caused by drivers [1]. Detecting and controlling driver behavior is an effective way to improve vehicle driving safety. Driving propensity is the driver’s attitude towards the actual traffic situation and the preference of the corresponding psychological decision or behavior value during the driving process, which can better reflect the relationship between driver factors and traffic accidents [2]. Intention recognition is the core part of the automotive active safety early warning system [3]. It is easy to overlook driving propensity as an important part of intention recognition. The effectiveness and accuracy of the early warning system need to be further improved. It is of great significance to improve the accuracy of safety early warning and reduce the occurrence of traffic accidents to refine the research on driving propensity of different drivers, conduct in-depth research on the identification methods of driving propensity and introduce driving propensity identification into the active safety driving assistance system of automobiles.

With the rapid development and wide application of electronic maps, there are map service providers represented by AutoNavi Maps in China, which provide users with free map location and driving navigation services. However, there are relatively few driving propensity studies based on navigation dynamic data. The massive real-time driving data has not been fully utilized. It is necessary to conduct in-depth research on driving propensity identification based on AutoNavi navigation dynamic data. Based on the Android development platform and AutoNavi Map SDK and API, a driving propensity identification system application is developed in this paper, which realizes the real-time collection, processing and storage of the driver’s dynamic data propensity identification features. Driving propensity can be accurately identified based on AutoNavi navigation data.

Wang Xiaoyuan et al. [4] proposed and defined the concept of driving propensity through systematic research on driver’s psychology and behavior. The influencing factors and performance characteristics of driving propensity were comprehensively analyzed from the dimensions of a human–vehicle environment. Song Yiqing [5] found that driver characteristics, vehicle characteristics, road conditions and weather conditions all had varying degrees of influence on driving propensity. Wang Mengsha [6] revealed the driving propensity transfer mechanism adapting to the time-varying law of vehicle grouping relationship by analyzing the vehicle grouping relationship in a multi-lane complex environment.

Wang Xiaoyuan et al. [7,8,9] adopted dynamic data acquisition systems such as vehicle-mounted LIDAR, multi-function speedometer, global positioning system and high-precision sensors. Time-varying data of a human–vehicle environment could be captured by designing real vehicle experiments, psychophysiological test experiments and driving simulation experiments. An exploratory study was carried out on the online representation and real-time identification of driver propensity using machine learning algorithms.

Driving propensity can be divided into dynamic driving propensity and static driving propensity. The dynamic driving propensity refers to the transient and changing driving propensity of the driver due to the influence of other factors such as changing traffic situation and road environment during the driving process. Static driving propensity refers to the relatively stable and profound driving habits formed by the driver in the past driving experience [4].

The static driving propensity is closely related to the driving style, which can reveal the static driving propensity to a certain extent [10]. Martinez et al. [11] believed that the driver’s driving style had an important impact on the vehicle’s energy management and driving safety. The research on driving style can be divided into three aspects. One is the way of collecting driving style data; the other is the selection of driving style characteristic parameters; the third is driving style recognition algorithm. Data used to identify driving propensity can be obtained in many different ways, such as driving simulator [12], Controller Area Network (CAN) [13,14], millimeter wave radar [15], vehicle camera [16,17], Global Positioning System (GPS) [18], on-board diagnostics (OBD) [15,19] and questionnaires [20,21]. The feature data used for driving style recognition can be divided into three categories. One is driver physical signals [22,23], such as steering angle, accelerator opening, gestures and other related signals; the other is driver physiological signals, such as ECG [24], EEG [25], EMG [26], etc.; the third is vehicle motion parameters, such as vehicle speed [27], acceleration [28,29,30], yaw angle [31], etc. Early research on driving style recognition algorithms was mostly based on rules and fuzzy logic methods [32]. However, the setting of the threshold is highly subjective and cannot be changed according to the dynamic data. In recent years, many researchers have carried out a lot of research on driving style recognition based on machine learning algorithms, such as clustering algorithm [33,34,35], Bayesian estimation algorithm [36], decision tree algorithm [37], support vector machine [38,39] and random forest [40,41]. Wang et al. [13] investigated a new framework for driving style analysis using raw driving patterns and Bayesian nonparametric methods using driving data collected by the Mobileye vision system and the vehicle CAN bus. Zhu et al. [14] used inertial navigation system, ranging system and vehicle CAN system to build a driving data acquisition platform. The platform can collect multi-dimensional data such as vehicle distance, vehicle relative angle, accelerator opening, master cylinder pressure, acceleration, steering angle, vehicle latitude and longitude. A typical driving style for the personalized adaptive cruise is studied. Long et al. [21] investigated the effectiveness of using a driving style scale to identify driving styles. The reliability and validity of the Chinese version of the Multidimensional Driving Style Inventory (MDSI) were verified by exploratory factor analysis and confirmatory factor analysis. Mohammadnazar et al. [33] utilized basic safety information generated by connected vehicles to quantify instantaneous driving behavior. Unsupervised machine algorithms were used to classify driver driving styles in different spatial environments and road types. The clustering results showed that there were differences in the driving styles of drivers. There were differences in the thresholds for aggressive and calm driving due to differences in the environment and road conditions. The percentage of people with aggressive driving styles was also higher on commercial streets than on highways and residential streets. Mantouka et al. [35] applied a two-stage K-means clustering method to detect drivers’ dangerous driving styles. Driving behavior data were obtained using speed and acceleration data from a smartphone. Aggressive and non-aggressive driving behaviors were detected by initial clustering and normal and dangerous driving styles of drivers were detected by secondary clustering. Suzdaleva et al. [36] studied the problem of online detection of driving style based on recursive Bayesian estimation of a mixture of regular and class components. On the premise of driving styles during driving, seven driving styles related to fuel economy were identified through an online estimation algorithm. The algorithm was also used to model and predict fuel consumption, speed, accelerator pedal position and gear selection. Wang et al. [39] used a semi-supervised support vector machine to classify driving styles while using a small amount of annotated driving data in order to solve the problem of manually annotating a large amount of driving data. Experiments showed that compared with SVM, the classification accuracy of semi-supervised SVM was improved by about 10%, which not only improved the classification performance in general, but also significantly reduces the need for prior data annotation.

At present, there is lack of driving propensity based on navigation dynamic data and the massive real-time driving data are not fully utilized. In the field of driving behavior research, there are relatively few applications of AutoNavi navigation data. Zhao et al. [42] developed the multinomial logit model (MNL) to explore the impact of factors, including day of the week, time of day, congestion level, traffic control devices and road conditions, on road safety risk levels in the interchange area of an urban expressway based on a large amount of aggregate driving behavior data obtained from AutoNavi software. The results showed that the factors that significantly influence risky roads include day of the week, number of lanes, congestion level (slow moving), traffic disturbance (with the merge or diverge within 500 m), type of advance guide sign system (three-level advance guide sign system) and complexity of diagrammatic guide signs (low or medium complexity). Bian et al. [43] investigated navigation prompt timing (NPT), navigation prompt message (NPM) and their combination in an audio navigation system on driving behavior on an urban expressway with five exits. The results showed that the driver’s psychological state and operation of the vehicle on the urban expressway were affected by the prompt timing and messages of the audio navigation system. Guo et al. [44] developed a traffic crash risk prediction model based on risky driving behavior and traffic flow. The data employed in their research were captured using the in-vehicle AutoNavigator software. The model accurately predicted 84.48% of the crashes, while its false alarm rate remained as low as 9.75%, which indicated that the traffic crash risk prediction model had high accuracy. By analyzing the relationship between traffic flow, risky driving behavior and crashes through partial dependency plots (PDPs), the impact of traffic flow and risky driving behavior variables on certain traffic crashes in the prediction model was determined.

Existing research on driving propensity and driving styles were mostly based on equipment such as driving simulators, millimeter-wave radars, global positioning systems and various sensors. Research studies based on driving simulator safety are easier to set up with different traffic scenarios. However, the real road conditions cannot be represented with the driving simulator and it is difficult to simulate the complexity and diversity of the real-world traffic environment. The equipment of the research based on the millimeter radar, global positioning system and various sensors is expensive, and the installation is relatively complicated. It is cumbersome to process the data, resulting in high identification cost and poor practicability. Aiming at the above problems, a dynamic data acquisition algorithm for AutoNavi navigation and a driving propensity identification method based on AutoNavi dynamic data and fruit fly optimization algorithm combined with GRNN (generalized regression neural network) are proposed in this paper. Finally, a driving propensity identification system is established based on the Android development platform and the AutoNavi map open platform. The real-time collection, processing and storage of the driver’s dynamic data during driving can be realized with the driving propensity system. The dynamic data acquisition method of AutoNavi is realized by constructing dynamic data acquisition application programs and designing data acquisition algorithms. Nine driving propensity characteristic datasets derived from time, speed and acceleration during navigation can be obtained through this method. The fruit fly optimization algorithm is used to iteratively optimize the smooth factor in GRNN to improve the prediction accuracy. The driving propensity identification system application is built based on the Android development platform and AutoNavi Map SDK and API. The application is integrated into the personal intelligent terminal to realize the real-time collection, processing and storage of the driver’s driving data and the accurate identification of the driving propensity during the navigation process. AutoNavi navigation data are applied to the identification of driving propensity for the first time to achieve the accurate prediction of a driver’s driving behavior and preferences. It is very important for preventing traffic accidents. This paper provides a novel idea for establishing a personalized vehicle active safety early warning system.

## 2. Materials and Methods

### 2.1. Participants

**Figure 1:**
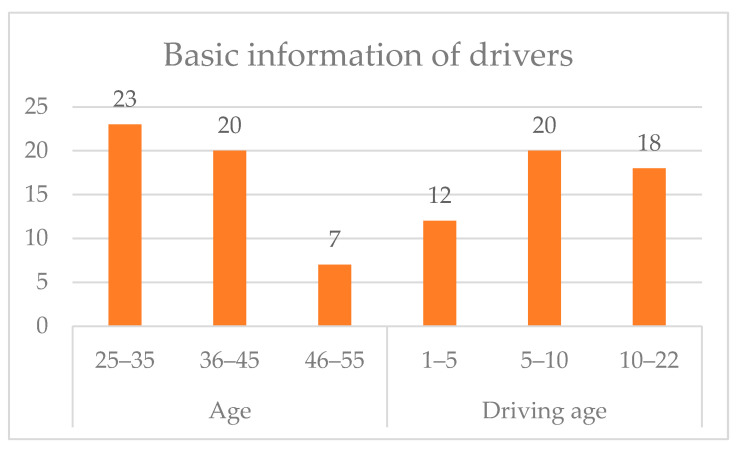
Basic information of the drivers.

A total of 50 drivers were organized to participate in the real vehicle experiment, of which the ratio of male to female was 8:2, the age distribution was between 25 and 55 years old and the driving experience was between 1 and 22 years old. The basic information of the drivers is shown in Figure 1.

### 2.2. Apparatus

**Figure 2:**
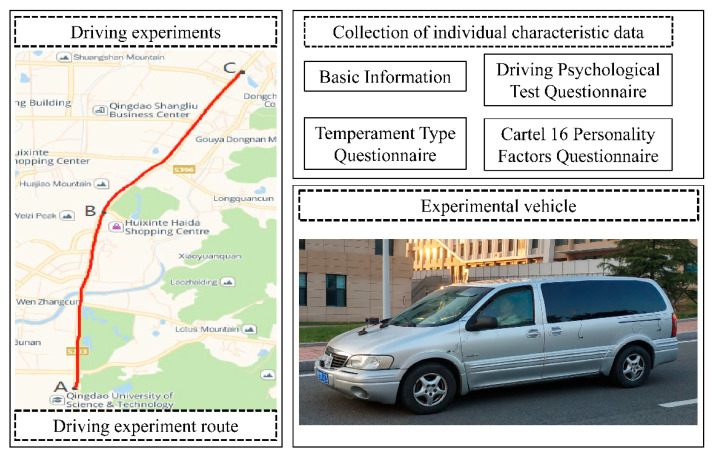
Experimental vehicle.

The experimental materials were temperament type questionnaire, cartel 16 personality factors questionnaire and driver psychological test questionnaire. These questionnaires are composed of questions representing driver’s psychophysiological characteristics and driving behavior characteristics, which has good content reliability and validity [4]. A GL8 experimental vehicle was used as the real vehicle platform, as shown in Figure 2. In this paper, only one smartphone can complete all data collection and realize real-time driving propensity identification.

### 2.3. Procedure

The real vehicle experiments were carried out during sunny weather and dry road conditions. The experimental road type was a general urban road and the route was along Songling Road, Laoshan District, Qingdao City, Shandong Province, China, as shown in Figure 2. The starting point was A, the end point was C and the way point was B. The whole journey was 9.45 km. The roads in section A–B were mostly commercial areas and residential areas. The traffic flow was large. The B–C section was mainly surrounded by factories. The traffic flow was relatively small. The specific time arrangement of the experiment was as follows: weekdays Tuesday morning peak 7:30–8:00, afternoon peak 15:30–16:00; weekday Wednesday morning peak 9:30–10:00, evening peak 18:30–19:00; non-working Saturday mornings 9:00–9:30 in the morning and 18:00–18:30 in the evening peak. The experiments lasted for four weeks.

Before the driving experiment, the basic information of each participant was recorded, including age, gender and driving experience. The participants were organized to fill out the driving psychological test questionnaire, the temperament type questionnaire and the cartel 16 personality factors questionnaire [4], as shown in Figure 2. The driving propensity type of the participants can be preliminarily determined by the questionnaire test results. The experimental vehicle was equipped with a debugged smartphone for dynamic data collection of AutoNavi navigation. Different participants drove in sequence on the experimental route. An experimental assistant was arranged to ensure the normal operation of the equipment during the experiment. At the end of each driver’s experiment, the dynamic driving data collection APP ended the navigation and automatically saved the real-time data to the mobile phone database. Due to the different experimental time periods, the experimental vehicle could obtain two sets of experimental data by going back and forth on the experimental route once.

### 2.4. AutoNavi Navigation Dynamic Data Acquisition Algorithm

The AutoNavi navigation dynamic data acquisition application program consists of a positioning module, navigation module, data acquisition module, data processing module and data storage module.

Map loading and basic map interaction functions can be realized by calling the AutoNavi map API in the positioning module. The secondary development is performed based on the AutoNavi positioning SDK, the location service and map application module of Android development platform. The real-time positioning function is achieved by applying for mobile phone positioning permission. The vehicle positioning data are called back through the positioning API interface. The positioning data can be obtained from this function with the data acquisition module. The navigation module implements basic navigation functions through callback path planning, navigation creation and real-time navigation. The vehicle driving data during the navigation process can be called back through the navigation API interface. The driver’s navigation driving data are obtained from this function with the data acquisition module. The data acquisition module is connected to the positioning module and the navigation module. Through the positioning and navigation data collection interface, the function is called back to obtain the real-time driving data of the driver, including vehicle latitude and longitude coordinates, vehicle speed, driving time and driving mileage. The data processing module receives and processes the real-time driving data transmitted by the data acquisition module. The characteristic data that better characterizes the driving propensity can be obtained with the driving data deduction algorithm, including travel time $T_{e}$, average speed $V_{ave}$, maximum speed $V_{\max}$, rapid acceleration times $N_{acc}$, rapid deceleration $N_{dec}$, normal acceleration time $T_{acc}$, normal deceleration time $T_{dec}$, average acceleration $A_{ave}$, and maximum acceleration $A_{\max}$. The data storage module receives and stores the dynamic data transmitted by the data acquisition module and the data processing module in real time.

A multi-dimensional driving propensity characteristic parameter acquisition algorithm is proposed in this paper. The specific acquisition algorithm of each characteristic parameter is as follows:

(1) Travel time $T_{e}$ acquisition algorithm

The travel time obtained in this paper is the effective travel time when the vehicle travels between the two endpoints of a certain navigation with non-zero driving speed. The driver’s driving propensity can be better characterized with this parameter. The travel time $T_{e}$ acquisition algorithm is implemented based on real-time monitoring of vehicle speed changes during navigation. After the navigation is turned on, accumulate the driving time when the speed is not zero every second. The system collection frequency is 1 Hz. The total travel time can be calculated until the end of the navigation. For the convenience of describing the algorithm, the introduction of intermediate variables is shown in Table 1.

**Figure 3:**
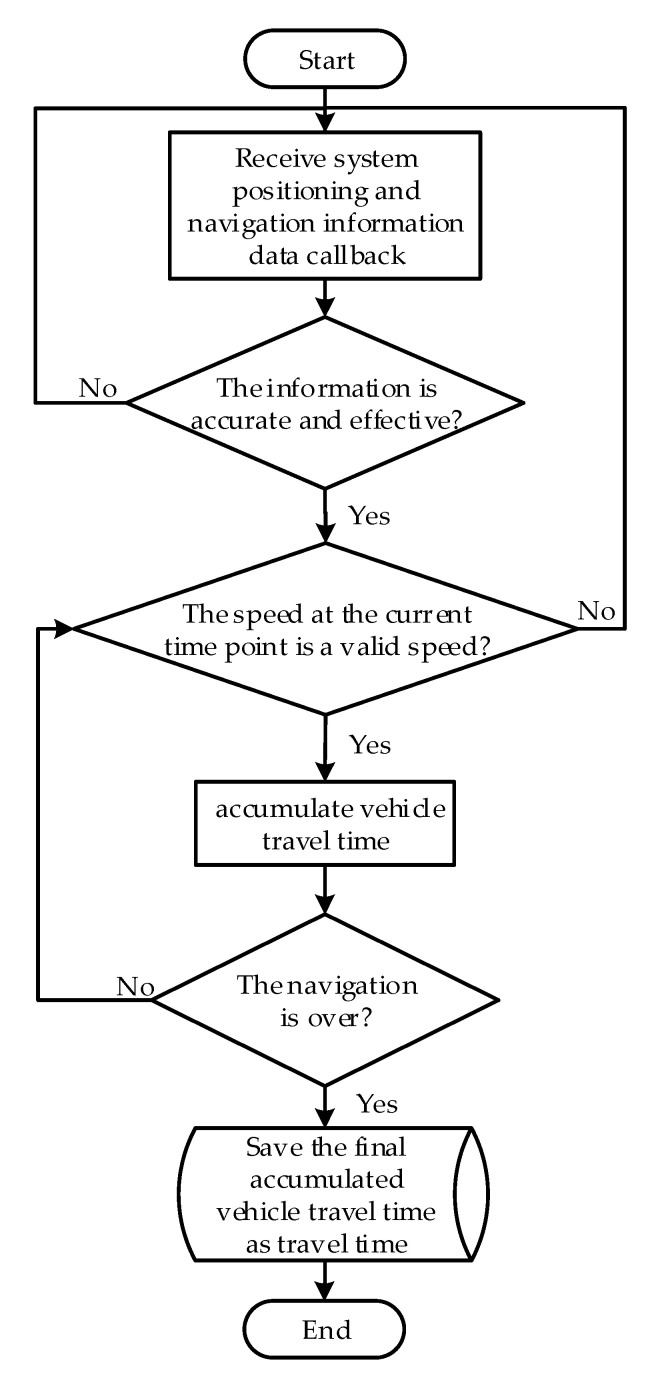
Flow chart of travel time $T_{e}$ acquisition algorithm.

The flow of travel time $T_{e}$ acquisition algorithm is shown in Figure 3.

(2) Average speed $V_{ave}$ and maximum speed $V_{\max}$ acquisition algorithm

The average speed $V_{ave}$ and maximum speed $V_{\max}$ obtained in this paper are the average effective speed and the maximum effective speed of the vehicle passing through the two endpoints of a certain section. That is, the average effective speed and the maximum effective speed of the vehicle within the effective travel time $T_{e}$. Average speed $V_{ave}$ and maximum speed $V_{\max}$ acquisition algorithm is implemented based on real-time monitoring of speed changes. After the navigation is turned on, detect every second when the speed is not zero. The system collection frequency is 1 Hz. Accumulate the speed corresponding to each second in the effective travel time $T_{e}$ while obtaining the effective travel time to get the effective speed sum $V_{sum}$. Divide it by the travel time to obtain the average speed $V_{ave}$. The calculation formula is shown in Equation (1). The maximum speed $V_{\max}$ is calculated by comparing the speed values $V_{n}$ before and after each second. The larger speed value $V_{n}$ is temporarily saved as the maximum speed $V_{\max}$. The comparison is continued until the end of the trip. The final maximum $V_{\max}$ speed is saved. The calculation formula is shown in Equation (2). For the convenience of describing the algorithm, the introduction of intermediate variables is shown in Table 2.

(1) $$V_{ave}=\frac{\sum_{T_{e}}^{n=1}V_{n}}{T_{e}}=\frac{V_{sum}}{T_{e}},\;(n=1,2,\cdots ,T_{e})$$

(2) $$V_{\max}=\left\{ \begin{array}{l} V_{n}\;\;\;V_{n}>V_{\max}\\ V_{\max}\;V_{n}\leq V_{\max}\; \end{array}\right.,\;(n=1,2,\cdots ,T_{e})$$

**Figure 4:**
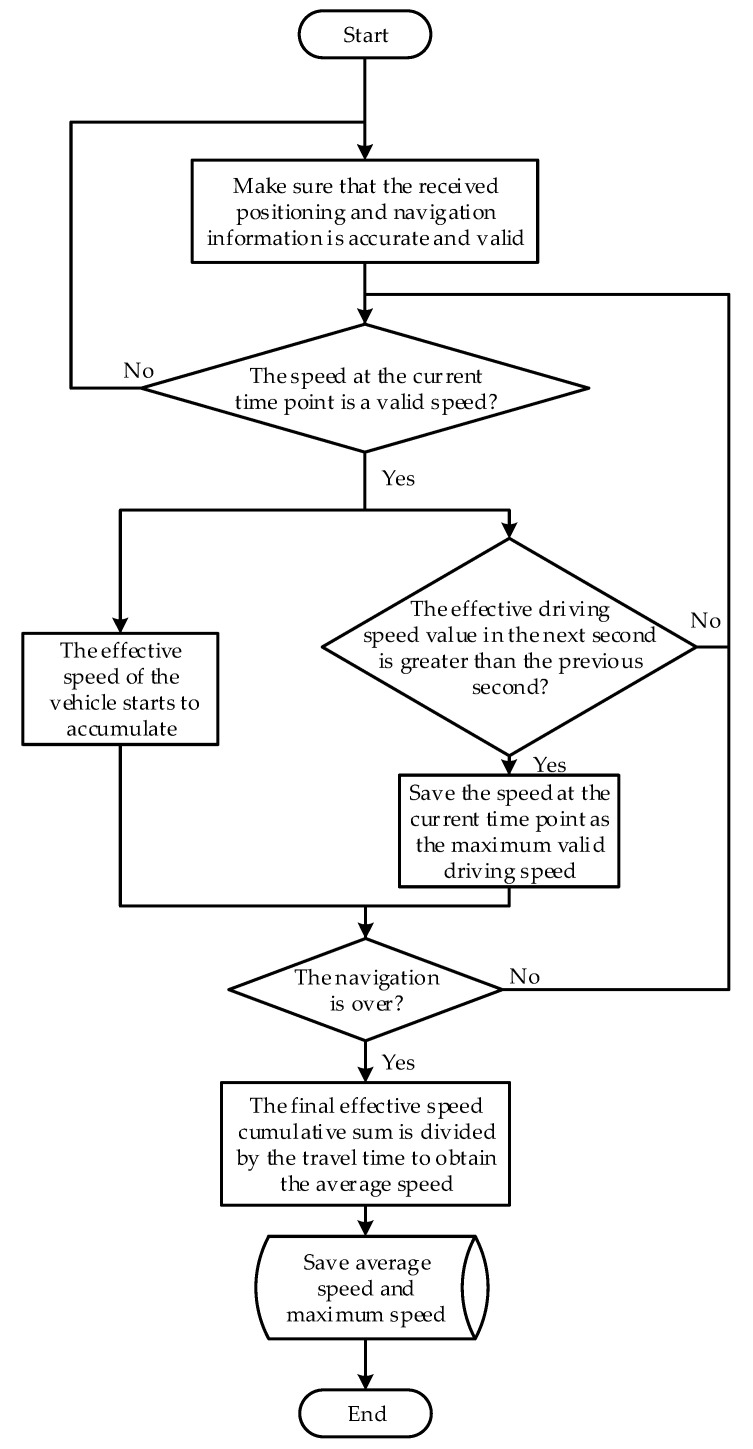
Flow chart of the average speed $V_{ave}$ and maximum speed $V_{\max}$ acquisition algorithm.

A flow chart of the average speed $V_{ave}$ and maximum speed $V_{\max}$ acquisition algorithm is shown in Figure 4.

(3) Rapid acceleration times $N_{acc}$, rapid deceleration $N_{dec}$, normal acceleration time $T_{acc}$ and normal deceleration time $T_{dec}$ acquisition algorithm

The rapid acceleration times $N_{acc}$, rapid deceleration $N_{dec}$, normal acceleration time $T_{acc}$ and normal deceleration time $T_{dec}$ obtained in this paper are the driving behavior events generated by the vehicle passing through the two endpoints of a certain navigation section. That is, the number of rapid acceleration behaviors, the number of rapid deceleration behaviors, the time of normal driving behavior, and the time of normal deceleration behavior generated by the vehicle within the effective travel time $T_{e}$. Rapid acceleration times $N_{acc}$, rapid deceleration $N_{dec}$, normal acceleration time $T_{acc}$ and normal deceleration time $T_{dec}$ acquisition algorithm is based on real-time monitoring of acceleration changes. The system collection frequency is 1 Hz. The acceleration can be calculated from the speed change per second using Equation (3).

(3) $$A_{n}=\frac{V_{n}-V_{n-1}}{1},\;(n=1,2,\cdots ,T)$$

When the acceleration $A_{n}$ is larger of less than different thresholds, sTime is recorded as the start time of the sudden acceleration (deceleration) behavior and the normal acceleration (deceleration) behavior event. At the same time, the system starts to monitor continuously. When the acceleration $A_{n}$ does not meet the threshold condition, it is recorded as the end time of the corresponding driving behavior event. Determine whether the duration meets the valid duration threshold of the driving behavior event. According to the relevant regulations of the traffic safety passage rules and the actual road test data, we selected the sudden acceleration threshold as 2.22 m/s^2^, the sudden deceleration threshold as −2.22 m/s^2^, the normal acceleration threshold as 0.45 m/s^2^, the normal deceleration threshold as −0.45 m/s^2^ and the effective duration threshold of the driving behavior event as 5 s. For the convenience of describing the algorithm, the introduction of intermediate variables is shown in Table 3.

**Figure 5:**
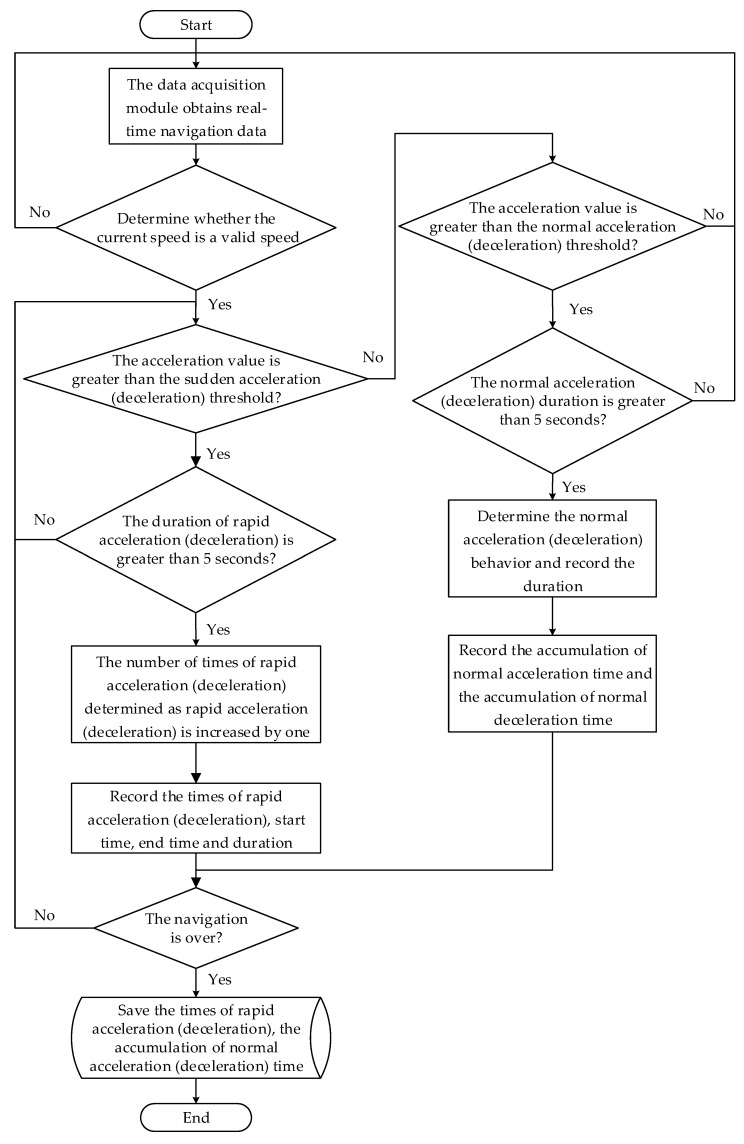
Flow chart of rapid acceleration times $N_{acc}$, rapid deceleration $N_{dec}$, normal acceleration time $T_{acc}$ and normal deceleration time $T_{dec}$ acquisition algorithm.

The flow chart of rapid acceleration times $N_{acc}$, rapid deceleration $N_{dec}$, normal acceleration time $T_{acc}$ and normal deceleration time $T_{dec}$ acquisition algorithm is shown in Figure 5.

(4) Average acceleration $A_{ave}$ and maximum acceleration $A_{\max}$ acquisition algorithm

The average acceleration $A_{ave}$ and maximum acceleration $A_{\max}$ obtained in this paper are the average acceleration and maximum acceleration generated by the vehicle during the normal acceleration time $T_{acc}$. The average acceleration $A_{ave}$ and maximum acceleration $A_{\max}$ acquisition algorithm is based on the real-time monitoring of acceleration changes. The system collection frequency is 1 Hz. The accumulated acceleration sum $A_{sum}$ can be obtained by accumulating the acceleration per second $A_{n}$ during the acceleration time $T_{acc}$. The average acceleration $A_{ave}$ can be obtained by the ratio of the accumulated acceleration sum $A_{sum}$ to the acceleration time $T_{acc}$, which can be calculated with Equation (4). At the same time, compare the acceleration value $A_{n}$ before and after each second in the acceleration time $T_{acc}$. The larger acceleration value is assigned to $A_{\max}$. If $A_{n}>A_{\max}$, $A_{\max}=A_{n}$; if $A_{n}<A_{\max}$, $A_{\max}$ keeps the original value unchanged. The comparison continues until the end of the stroke, saving the maximum acceleration $A_{\max}$. The calculation formula is shown in Equation (5). For the convenience of describing the algorithm, the introduction of intermediate variables is shown in Table 4.

(4) $$A_{ave}=\frac{\sum_{T_{acc}}^{n=1}A_{n}}{T_{acc}}=\frac{A_{sum}}{T_{acc}},\;(n=1,2,\cdots ,T_{acc})$$

(5) $$A_{\max}=\left\{ \begin{array}{l} A_{n}\;\;\;A_{n}>A_{\max}\\ A_{\max}\;A_{n}\leq A_{\max}\; \end{array}\right.,\;(n=1,2,\cdots ,T_{acc})$$

**Figure 6:**
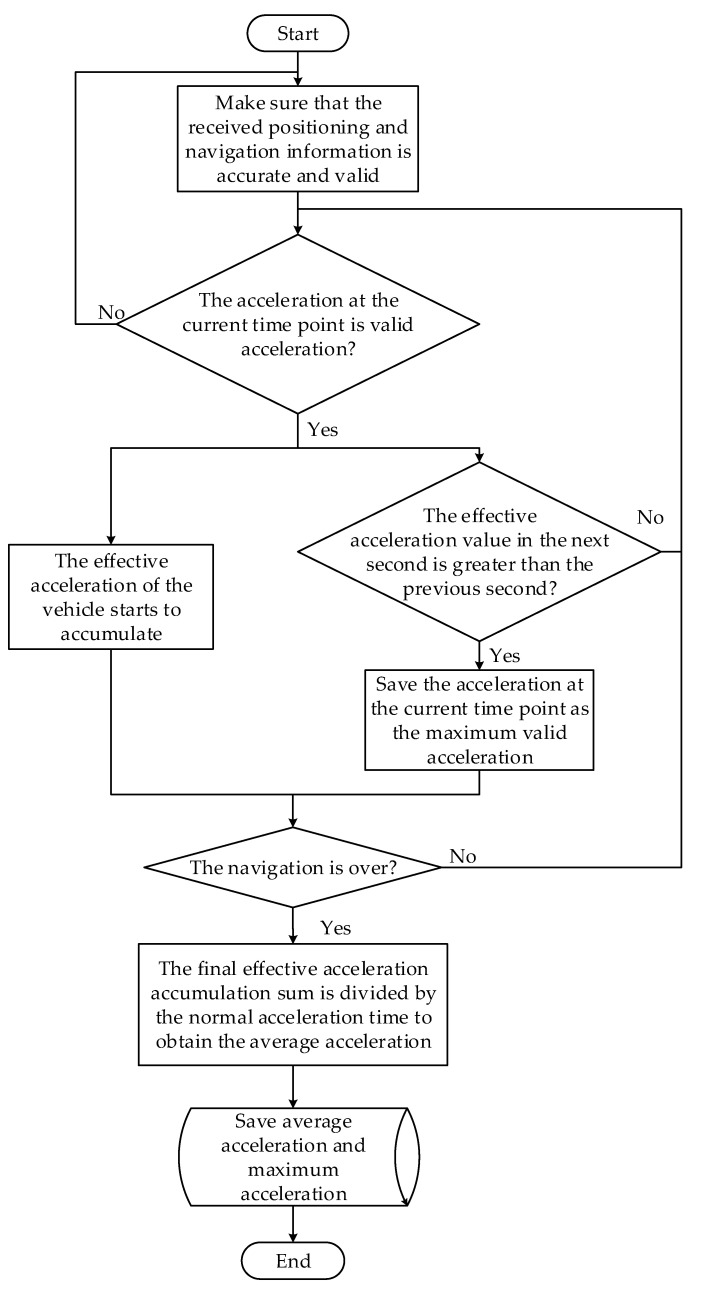
Flow chart of the average acceleration $A_{ave}$ and maximum acceleration $A_{\max}$ acquisition algorithm.

The flow chart of the average acceleration and maximum acceleration $A_{\max}$ acquisition algorithm is shown in Figure 6.

## 3. Results and Discussion

### 3.1. Data

The experimental data collected in this paper contain 12 driving characteristic variables, as shown in Table 5.

Some data are shown in Table 6.

After the experiment, the questionnaire test results of each driver in the experimental sample are counted, and the preliminary prediction results of each driver’s driving propensity are recorded. The driver’s driving behavior responses when faced with different traffic situations and road environments can be viewed through video playback, including the driver’s operational response, facial expressions, and driving propensity. The driving propensity can be comprehensively determined according to the driver’s driving behavior responses and the preliminary prediction result of the driving propensity. The preliminary judgement result of driving propensity is shown in Table 7.

### 3.2. Driving Propensity Feature Extraction Method

The data in this paper are collected through real vehicle experiments in the real road environment. This results in a lot of data noise, which causes many fluctuations in the collected raw data and affects the model training. Thus, a sliding mean filter is selected to process raw data, such as velocity and acceleration. The mean filter expression is as follows:

(6) $${\bar{X}}_{n}=\frac{1}{M}\sum_{i=0}^{M-1}X_{n-i}$$

Among this, M is the sliding filter window size. $X_{n-1}$ is the n−i original data. The sliding filter window size M has a great impact on the filtering effect. According to the acquisition frequency of the original data (10 Hz), the value of M is selected as 5 to perform smoothing filtering on the original data.

By analyzing the characteristic parameters of driving propensity, the data size of each characteristic parameter is quite different, which can affect the target result. The data need to be normalized according to Equation (7).

(7) $$a=\frac{a^{\prime }-\min a^{\prime }}{\max a^{\prime }-\min a^{\prime }}$$

Among this, d is the original data and a is the normalized value of input data d.

The driving propensity feature parameters collected in this paper contain multiple dimensions. Although the higher the dimension of the feature data, the better it can represent the driving propensity. However, there is a correlation between high-dimensional feature data, which will cause data redundancy. The principal component analysis (PCA) algorithm is used to reduce the dimension of the characteristic parameter set of driving propensity. The main factors in the driving propensity feature parameter set are extracted to obtain a set of principal component feature vectors that can represent each driving propensity type. Each principal component is linearly uncorrelated with each other. The explanation of the total variance of each principal component is shown in Table 8.

It can be seen from Table 8 that the total variance explained by the first five principal components reaches 88.311%.

**Figure 7:**
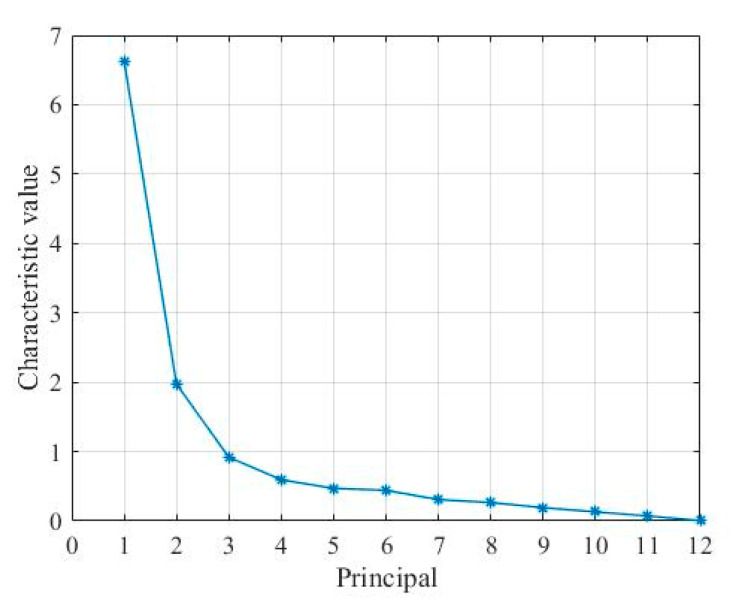
Characteristic value of each component.

Generally, according to the requirement that the cumulative contribution rate is greater than 85%, the first five principal components can fully characterize the changing characteristics of driving propensity. The characteristic values corresponding to the interpretation of the total variance of each principal component are shown in Figure 7. The characteristic value of each principal component also shows that the information contained in the first five principal components can better characterize the changing characteristics of driving propensity. Combined with the cumulative contribution rate and component eigenvalues of the characteristic parameters of each driving propensity, the first five principal components are the characteristic variables for driving propensity identification.

The scores of the first five principal components are given in Table 9. These five principal components are linear combinations of 12 feature variables. The scores of the principal components are used as the input in the driving propensity identification model.

### 3.3. Driving Propensity Recognition Model Based on FOA-GRNN

**Figure 8:**
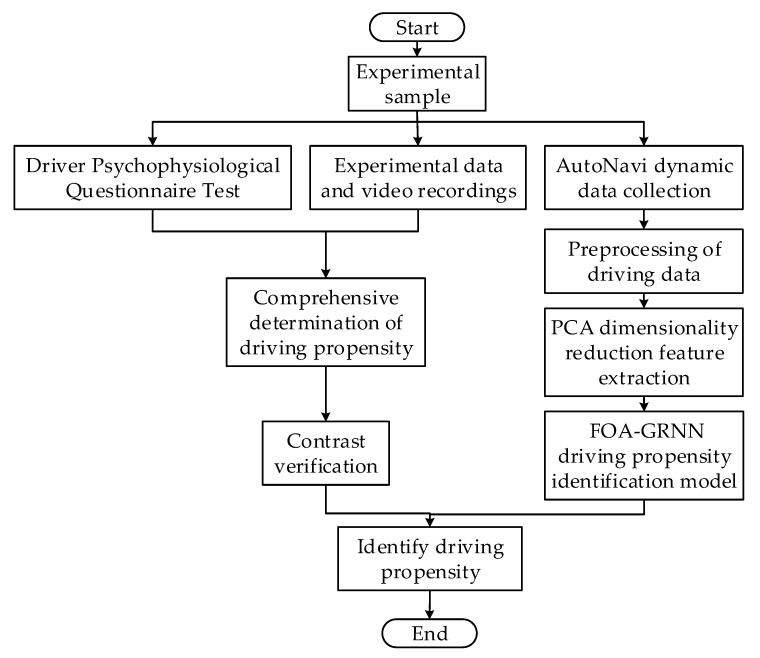
Flow chart of the driving propensity identification method.

A driving propensity identification method based on AutoNavi dynamic and FOA-GRNN is proposed in this paper. The process is shown in Figure 8.

Driving propensity is a dynamic measurement of the behavioral preference characteristics of car operators during driving. The types of driving propensity can be divided into aggressive, normal and conservative. The generalized recurrent neural network (GRNN) proposed by Donald F. Specht is a variant of radial basis neural network [45]. GRNN is a neural network that uses the probability density function to predict the output of the input data. It has strong learning ability and nonlinear mapping ability. It can still achieve good classification results with a small number of samples. Therefore, GRNN is chosen to dynamically identify the driving propensity.

The basic structure of GRNN includes a four-layer network of input layer, pattern layer, summation layer and output layer [46]. The input of the model is $X={\left[x_{1},x_{2},\cdots ,x_{n}\right]}^{T}$. The output of the model is $Y={\left[y_{1},y_{2},\cdots ,y_{n}\right]}^{T}$ and y={0,1,2}.

**Figure 9:**
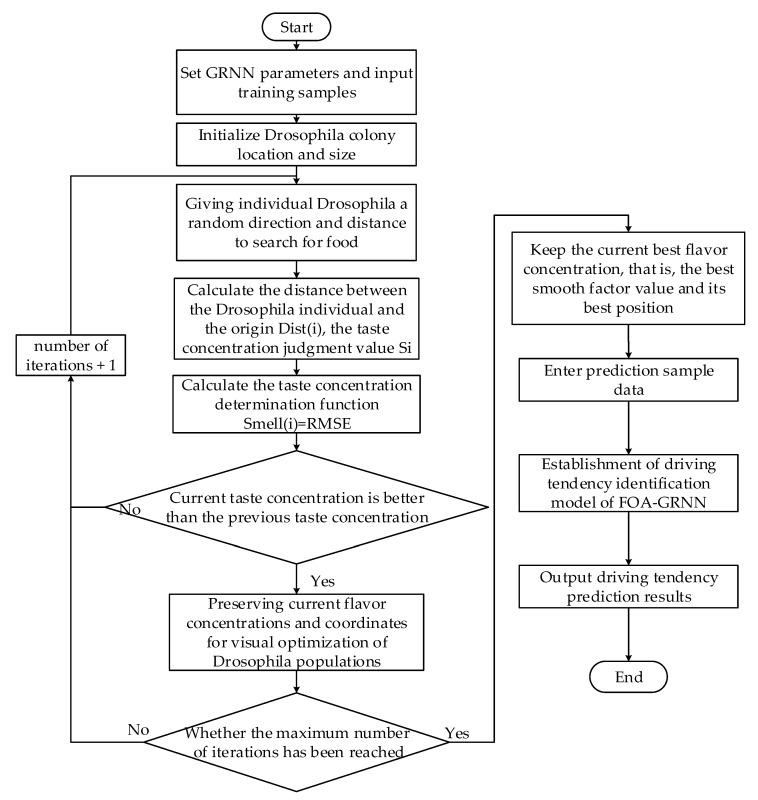
Flow chart of the FOA optimization GRNN model.

The fruit fly optimization algorithm (FOA) is proposed by Wenchao Pan in 2011 [47]. The olfactory and visual characteristics of fruit flies is cleverly used to search for food for iterative optimization with FOA algorithm. The principle of FOA algorithm has the advantages of simple principle and fast convergence speed [48]. The basic principle of FOA algorithm can be divided into two stages: drosophila uses smell to search for food and vision is used to observe and determine the location of the food, and then fly towards that location. The prediction accuracy of GRNN is greatly affected by the smooth factor and the drosophila optimization algorithm is an effective method to optimize the smooth factor [49]. The smooth factor value is optimized by the fruit fly optimization algorithm. The specific optimization idea is to adjust the smooth factor value to the optimum by using the mechanism of drosophila olfactory random foraging and visual search for the highest odor concentration position. The root mean square error (RMSE) between the predicted value and the actual value of the network output driving propensity is minimized through iterative optimization. The corresponding drosophila taste concentration value reaches the optimal value as the minimum value of RMSE. That is, the smooth factor in the generalized regression neural network obtains the optimal value, and it is input into the GRNN model. The driving propensity identification model based on the drosophila optimization algorithm to optimize GRNN is shown in Figure 9. The specific implementation steps are as follows.

Step 1: Input the driving propensity feature variable set and divide the training set and test set. Set GRNN parameters and input the training samples.

Step 2: Randomly initialize the fly position $\left(Init\_X_{0},Init\_Y_{0}\right)$. Set the population size (Sizepop) and the maximum number of iterations (Maxgen).

Step 3: Give individual drosophila a random direction and distance to search for food by smell $\left(X_{i},Y_{I}\right)$.

(8) $$X_{i}=X_{0}+Rand()$$

(9) $$Y_{i}=Y_{0}+Rand()$$

Step 4: Determine the distance Dist(i) between the coordinates of each drosophila and the origin, and then calculate the taste concentration judgment value $S_{i}$, which will be used as a smoothing factor for GRNN

(10) $$Dist(i)=\sqrt{\left(X_{i}^{2}+Y_{i}^{2}\right)}$$

(11) $$\sigma =S_{i}=\frac{1}{Dist(i)}$$

Step 5: Substitute the taste concentration judgment value into the taste con-centration judgment condition (if $S_{i}$ < 0.001, $S_{i}$ = 1, if $S_{i}$ > 0.001, $S_{i}$ = $S_{i}$). Bring the smooth factor value σ = $S_{i}$ into the GRNN model to obtain the taste concentration Smell(i) of the individual location of the drosophila. In this paper, the root mean square error RMSE of the driving propensity prediction obtained by the GRNN model is used as the taste concentration Smell(i).

(12) $$Smell(i)=\sqrt{\frac{\sum_{i=1}^{M}{\left(y_{i}-\hat{y_{i}}\right)}^{2}}{M}}$$

Step 6: Find the location of the individual with the lowest taste concentration Smell(i) in the drosophila population.

(13) [bestSmellbestIndex]=min(Smell(i))

Step 7: Determine whether the taste concentration is better than the previous taste concentration. If yes, go to Step 8; if not, return to Step 3 to continue iterative optimization.

Step 8: Keep the optimal taste concentration value and the corresponding drosophila individual coordinates, and the drosophila will use vision to fly to this location.

(14) $$\begin{array}{l} Smellbest=bestSmell\\ X_{best}=X(bestIndex)\\ Y_{best}=Y(bestIndex) \end{array}$$

Step 9: Determine whether the maximum number of iterations has been reached, and if so, save and output the optimal flavor concentration, with the optimal smooth factor as σ. Establish the driving propensity identification model of FOA_GRNN.

Step 10: Identify the driving propensity of the input driver prediction sample data.

In this paper, a driving propensity identification model is established based on FOA-GRNN. The experimental data of 30 drivers were selected from the experimental samples for the training and testing of the model. Among them, we selected 1200 sets of experimental data of 10 drivers of aggressive type, ordinary type and conservative type, respectively, and divided them into a training set and test set according to the ratio 8:2. In order to further verify the trained driving propensity identification model, the experimental data of another 20 drivers in the experimental sample were input into the model to verify the recognition accuracy of the model for each type of driving propensity.

In this study, MATLAB 2019a was used for model training simulation experiments. The initial position interval of the drosophila group was set to [0,1], the size of the drosophila group was 10 and the flight direction and distance interval of the drosophila group to search by smell was [−10,10], the maximum number of iterative optimizations of the drosophila population is 200. In the divided training set samples, the first five principal components selected by principal component analysis are used as the input vector for driving propensity identification to train the model.

**Figure 10:**
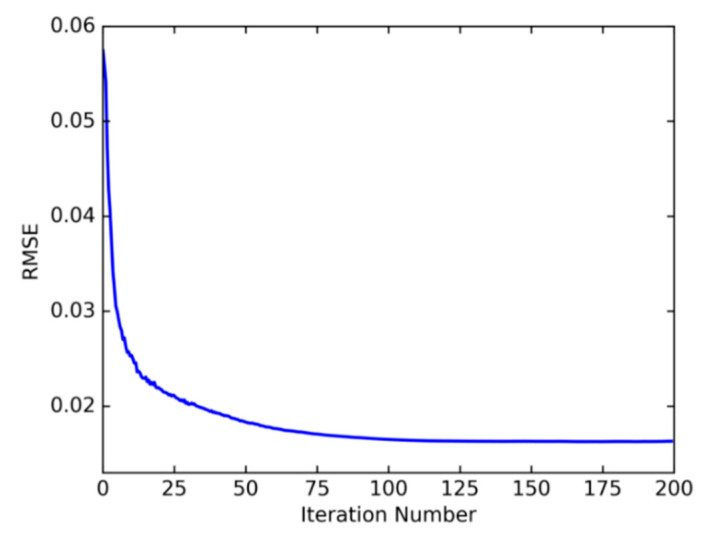
Root mean squared error convergence.

The convergence effect of the root mean square error (RMSE) of driving propensity prediction after 200 iterations of optimization is shown in Figure 9. From the convergence of RMSE in Figure 10, it can be seen that the effect of early iteration of FOA is more obvious and the update of flavor concentration is faster. In the iterative optimization process, the RMSE begins to converge in the 105th generation. At this time, the minimum error value is 0.016, that is, the minimum flavor concentration value is 0.016 and the optimal smooth factor σ is 0.062. At this time, the position of the drosophila population is (65.4529, −125.8327).

At this time, the optimal smooth factor σ = 0.062 is substituted into the GRNN network model and the test samples are input into the optimized driving propensity identification model. Limited to the length of the article, only the test results of the aggressive samples are shown here, as shown in Table 10, where 0 represents the aggressive type, 1 represents the ordinary type and 2 represents the conservative type.

The overall accuracy of the driving propensity identification model based on FOA-GRNN can reach 94.17%, which has high identification accuracy and can effectively identify various driving propensity types. It can be shown from Table 11 that the GRNN optimized by FOA iteratively optimizes the value of the smooth factor and has a good predictive ability, and the established FOA-GRNN identification model has a good identification effect on the driving propensity.

In order to further verify the identification accuracy of the established FOA-GRNN driving propensity identification model for each driver’s driving propensity, the experimental data corresponding to the other 20 drivers in the real vehicle experimental sample were selected for model verification. The final verification results are shown in Table 11. The verification results show that the driving propensity identification model based on FOA-GRNN has an accuracy rate of about 95% for aggressive and conservative drivers, and more than 92% for ordinary drivers, which shows that it has high identification accuracy for each driver’s driving propensity. Since the characteristics of aggressive and conservative drivers are more obvious than ordinary drivers, both aggressive and conservative drivers are better than ordinary drivers in terms of model identification accuracy 

As a comparison, the single generalized regression neural network (GRNN) and the BP neural network (back propagation neural network, BPNN) were used to process the same data samples to establish a driving propensity identification model and test the performance of two models. The identification accuracy is compared with the accuracy of the FOA-GRNN identification model.

The prediction accuracy of GRNN is greatly affected by the smooth factor σ, so this paper randomly selects 10 groups of smooth factors for testing and obtains the accuracy of the identification model. The test results are shown in Table 12.

It can be seen from Table 12 that the accuracy rate of the GRNN driving propensity identification model is the highest at 89.2%. The identification effect, which can be greatly affected by the smooth factor, is not ideal. The smooth factor of the GRNN model needs to be manually adjusted to find the best identification effect. The optimization process is cumbersome. Compared with the single GRNN identification model, the overall accuracy of the FOA-GRNN identification model proposed in this paper is improved by 5~10%, which has a better stability.

A BPNN driving propensity identification model which uses a 5-7-3 neural network structure is constructed according to the number of nodes in the input layer and output layer. The quasi-Newton method (trainbfg) is selected as the training function, the sigmoid function is the hidden layer transfer function, and the softmax function is selected as the transfer function for the output layer. Many parameters of the BP neural network need to be adjusted. The learning rate and training accuracy have a great influence on the algorithm. The three groups of learning rate (lr) are set to 0.01, 0.05 and 0.1. The three groups of training accuracy (goal) are set to 0.1, 0.01 and 0.001, and the maximum number of training times is 500. The test results are shown in Table 13.

It can be seen from Table 13 that the highest accuracy rate of the BPNN driving propensity identification model is 91.3%. By manually adjusting the parameters of the BPNN network, the model can achieve a good identification effect. However, many parameters of BPNN need to be set and the training process is cumbersome, which cannot guarantee the best recognition effect of the model. The comparison results show that the driving propensity identification accuracy of the GRNN model is the lowest. The identification accuracy of the BPNN model can achieve good results, but it has slow learning speed and too many parameters, and continuous tuning is required. The generalized regression neural network optimized by the fruit fly optimization algorithm proposed in this paper has the advantages of simple optimization process, high accuracy and good stability. It has higher identification accuracy and better stability in driving propensity identification.

### 3.4. Real Vehicle Experimental Test

**Figure 11:**
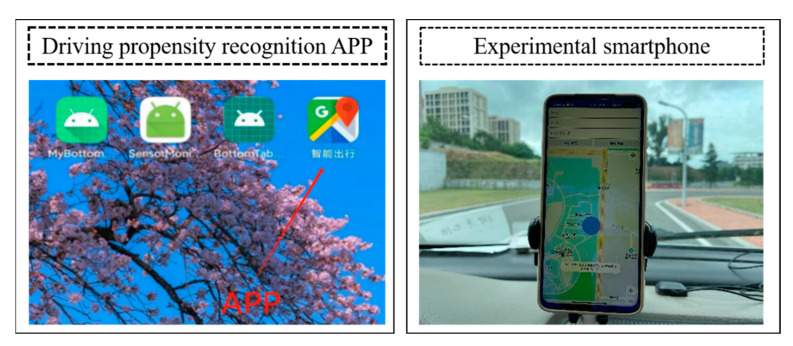
Driving propensity recognition APP and experimental smartphone.

Based on the Android development platform and AutoNavi SDK, a driving propensity identification system APP suitable for Android smartphones is developed in this paper, as shown in Figure 11. The smartphone is used as the carrier of the driving propensity identification system. The Android smartphone used in this experiment is the Redmi K30i mobile phone. The hardware configuration is as follows: the CPU is Qualcomm Snapdragon 765 G, the main frequency is 2.4 GHz, the eight-core, the running memory is 6 GB and the body memory is 128 GB.

In order to test the validity and reliability of the driving propensity identification system APP, three types of experiments are designed in this paper. The trained FOA-GRNN driving propensity identification model and the single GRNN and BPNN driving propensity identification models are imported into the system driving propensity identification module, and then the packaged driving propensity identification model is imported. The identification system APP is installed in the smartphone and fixed on the experimental vehicle. Twenty experimenters were selected to carry out driving experiments in sequence and the driving propensity identification system APP was used to collect, process and identify the driving propensity of the driver’s dynamic data during the navigation process. The specific experiments were as follows:

**Experiment 1:** The driving propensity identification system based on the FOA-GRNN model was used to conduct a real vehicle experimental test and the experimenters conducted 240 groups of experiments, including 80 groups of aggressive type, normal type and conservative type. The experimental results are shown in Table 14. 

By calculating and analyzing the identification results in the above table, various evaluation indicators of the driving propensity identification system were obtained as shown in Table 15. 

It can be seen from Table 15 that the system model has high identification accuracy and recall rate. The characteristics of aggressive and conservative drivers are more obvious than normal drivers. Therefore, in terms of precision, recall rate and comprehensiveness, both aggressive and conservative drivers are better than normal drivers.

**Experiment 2:** The driving propensity identification system based on the GRNN models was used to conduct the real vehicle experimental test and the experimenters conducted 240 groups of experiments, including 80 groups of aggressive type, ordinary type and conservative type. The experimental results are shown in Table 16.

By calculating and analyzing the identification results in the above table, various evaluation indicators of the driving propensity identification system were obtained as shown in Table 17.

**Experiment 3:** The driving propensity recognition system based on BPNN was used to conduct the real vehicle experimental test and the experimenters conducted 240 groups of experiments, with 80 groups for the aggressive type, the ordinary type and the conservative type, respectively. The experimental results are shown in Table 18.

By calculating and analyzing the identification results in the above table, various evaluation indicators of the driving propensity identification system were obtained as shown in Table 19.

**Figure 12:**
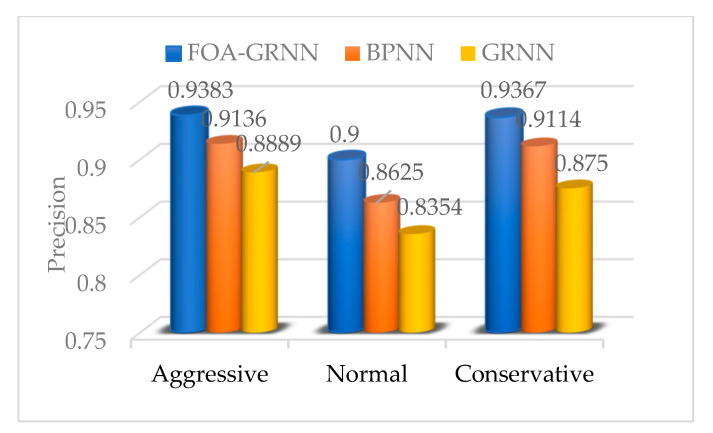
Precision comparison of the FOA-GRNN, BPNN and GRNN identification models.

**Figure 13:**
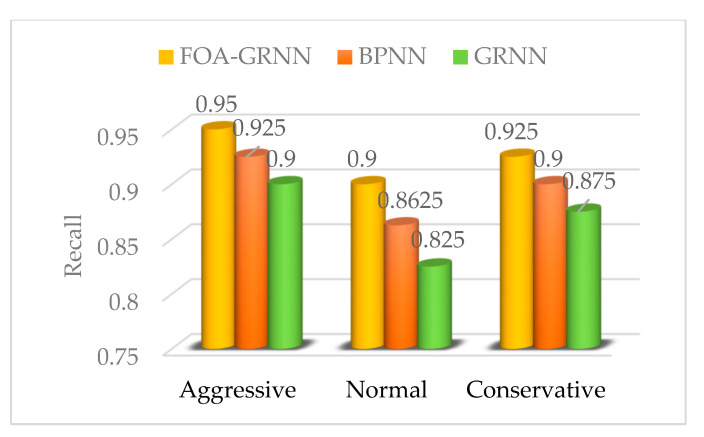
Recall comparison of the FOA-GRNN, BPNN and GRNN identification models.

**Figure 14:**
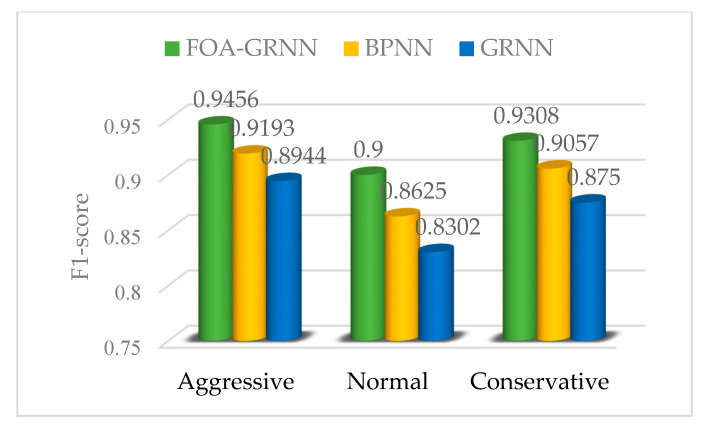
F1 score comparison of the FOA-GRNN, BPNN and GRNN identification models.

The performance indicators of the driving propensity identification system based on the FOA-GRNN model, GRNN model and BPNN model are shown in Figure 12, Figure 13 and Figure 14. Compared with the driving propensity identification system based on the GRNN model, the accuracy of the driving propensity identification system based on the FOA-GRNN model is at least 5% higher, and the accuracy of the driving propensity identification system is also improved compared to the BPNN model. In terms of precision, recall and F1 score, the driving propensity identification system based on the FOA-GRNN model has better performance indicators for aggressive, normal and conservative drivers than the other two systems. The identification effect and the system stability are higher than the other two systems. Although the identification accuracy of the system model in the real vehicle test is lower than training in MATLAB, it still achieves good identification accuracy. The generalization ability of the system model is strong, which verifies the effectiveness and practicability of the system.

## 4. Conclusions

Driving propensity is the driver’s attitude towards the real traffic situation and the preference of the corresponding decision-making or behavior value in the process of driving, which can better reflect the relationship between the driver’s factors and traffic accidents.

In the existing driving propensity-related research, the driving data collection equipment is expensive, the installation is complicated and the experimental data processing is cumbersome, resulting in high costs and poor practicability for the identification of driving propensity. There are relatively few studies that do not take full advantage of the vast amount of real-time driving data. A driving propensity identification method based on AutoNavi navigation dynamic data and FOA-GRNN is proposed in this paper and a driving propensity identification system based on the Android development platform and AutoNavi map open platform is established. Starting from the realization of personalized automotive active safety assistance system, in-depth research on the identification method of driving propensity was conducted. The specific research results are as follows:

(1) The dynamic data collection method of AutoNavi Navigation. A dynamic data acquisition method for AutoNavi Navigation is proposed in this paper. A dynamic data collection application based on the Android development platform and AutoNavi map API and SDK is developed. The data such as time, speed and acceleration are collected through AutoNavi API. The algorithm which can collect nine kinds of driving propensity characteristic parameters is designed, which realizes the real-time collection, processing and storage of driver characteristic data in the process of navigation and driving. This makes it possible to accurately identify the driving propensity based on the dynamic data of AutoNavi.
(2) An experimental framework suitable for driving propensity research. Starting from the consideration of the driver’s own factors, the driving propensity is preliminary judged through the authoritative test questionnaire. The reliability and validity of the test results are analyzed in this paper. Combined with the observation during the driver’s experiment and the video playback after the experiment, the driving propensity of the driver is comprehensively determined. The feasibility of the AutoNavi’s dynamic data acquisition program and the effectiveness of the acquisition algorithm are verified by the real vehicle experimental data.
(3) Feature parameter extraction of driving propensity. Considering the computational timeliness of the driving propensity model, the multidimensional data collected from the real vehicle experiments are processed for dimensionality reduction. The principal component analysis algorithm is selected to reduce the dimension of driving data and filter out redundant features. The feature data that contributes the most to various driving propensity is extracted and finally the feature parameters that better represent the driving propensity are obtained.
(4) Driving propensity identification model. Combined with the fruit fly optimization algorithm and generalized regression neural network, a driving propensity identification model based on FOA-GRNN is proposed. The model is trained, tested and verified by using the driving propensity feature variable set. The results show that the FOA-GRNN model proposed in this paper can realize the accurate identification of driving propensity and achieve better results for the identification of various driving propensity types. Compared with the GRNN and BPNN models, it is proved that the FOA-GRNN model has better stability and higher identification accuracy.
(5) Driving propensity identification system. By analyzing the functional requirements of the system, the overall framework of the system is determined and a modular system construction method is designed. Based on the Android development platform and AutoNavi map API and SDK, the driving propensity identification system is constructed. It is verified with the real vehicle experimental test that the functional modules of the system can operate stably, the overall performance of the model is better and the driving propensity can be accurately identified. The construction of this system can provide a new idea for the establishment of a human-centered safety-assisted driving system, which has certain practical significance.

## Figures and Tables

**Table 1:** Intermediate variables of travel time acquisition algorithm.

Name	Symbol	Data Type	Unit	Variable Description
Journey time	$T_{e}$	int	s	Sum of valid travel time within the navigation segment
Driving speed	$V_{n}$	double	m/s	Effective speed of the vehicle at the current time point
Driving time	T	int	s	Effective time of the current vehicle travel

**Table 2:** Intermediate variables of average and maximum velocity acquisition algorithm.

Name	Symbol	Data Type	Unit	Variable Description
Driving speed	$V_{n}$	double	m/s	Effective speed of the vehicle at the current time point
Sum of speed	$V_{sum}$	double	m/s	Total effective driving speed in the navigation section
Average speed	$V_{ave}$	double	m/s	Ratio of the total effective driving speed to the travel time in the navigation segment
Maximum speed	$V_{\max}$	double	m/s	Maximum effective speed in the navigation section

**Table 3:** Number of rapid acceleration and rapid deceleration times and duration of normal acceleration and normal deceleration to obtain the intermediate variables of the algorithm.

Name	Symbol	Data Type	Unit	Variable Description
Rapid acceleration times	$N_{acc}$	int	n	Number of sudden acceleration times in the navigation section
Rapid deceleration times	$N_{dec}$	int	n	Number of sudden deceleration times in the navigation section
Normal acceleration time	$T_{acc}$	int	s	Total duration of normal acceleration behavior in the navigation segment
Normal deceleration time	$T_{dec}$	int	s	Total duration of normal deceleration behavior in the navigation segment
Acceleration	$A_{n}$	float	${m/s}^{2}$	Effective acceleration of the vehicle at the current time point
Start time	sTime	double		Start time of the driving behavior event
End time	eTime	double		End moment when the driving behavior event occurred
Duration	time	double		Duration of driving behavior time

**Table 4:** Intermediate variables of average acceleration and maximum acceleration acquisition.

Name	Symbol	Data Type	Unit	Variable Description
Average acceleration	$A_{ave}$	float	${m/s}^{2}$	Average vehicle acceleration during normal acceleration time
Maximum acceleration	$A_{\max}$	float	${m/s}^{2}$	Maximum acceleration of the vehicle during normal acceleration time
Sum of acceleration	$A_{sum}$	float	${m/s}^{2}$	Accumulated sum of acceleration per second during normal acceleration time

**Table 5:** Driving characteristic variables and representation symbols.

Name	Symbol	Name	Symbol
Age (year)	a	Rapid acceleration times	$N_{acc}$
Driving age (year)	$D_{A}$	Rapid deceleration times	$N_{dec}$
Gender	G	Acceleration time	$T_{acc}$
Journey time (s)	T	Deceleration time	$T_{dec}$
Average speed (m/s)	$V_{ave}$	Average acceleration	$A_{ave}$
Maximum speed (m/s)	$V_{\max}$	Maximum acceleration	$A_{\max}$

**Table 6:** Partial experimental data.

Number	a	$D_{A}$	${G\;}^{1}$	T	$V_{ave}$	$V_{\max}$	$N_{acc}$	$N_{dec}$	$T_{acc}$	$T_{dec}$	$A_{ave}$	$A_{\max}$
01	37	12	0	806	11.09	20.56	6	4	84	75	0.435	1.943
	37	12	0	800	11.20	20	5	2	84	73	0.479	2.012
	…	…	…	…	…	…	…	…	…	…	…	…
	37	12	0	802	11.17	19.72	6	5	87	75	0.478	1.735
02	34	10	1	759	12.05	21.11	7	5	95	76	0.715	2.091
	34	10	1	754	12.1	21.39	6	5	89	72	0.568	2.423
	…	…	…	…	…	…	…	…	…	…	…	…
	34	10	1	747	11.75	20	7	4	91	74	0.672	2.271
03	26	6	1	691	13.02	21.11	13	9	95	77	0.763	2.792
	26	6	1	698	12.94	23.33	11	10	90	75	0.892	2.878
	…	…	…	…	…	…	…	…	…	…	…	…
	26	6	1	702	12.89	21.94	8	8	92	78	0.781	2.973
…	…	…	…	…	…	…	…	…	…	…	…	…
23	40	12	1	707	12.81	22.5	9	10	97	78	0.892	2.562
	40	12	1	698	12.94	23.6	11	6	95	82	0.831	3.261
	…	…	…	…	…	…	…	…	…	…	…	…
	40	12	1	702	12.89	22.4	10	7	92	78	0.785	3.178
24	36	10	1	818	10.82	19.72	5	5	86	83	0.472	1.738
	36	10	1	808	11.05	18.89	6	3	93	87	0.418	1.351
	…	…	…	…	…	…	…	…	…	…	…	…
	36	10	1	823	10.75	18.89	4	1	83	88	0.378	1.943
…	…	…	…	…	…	…	…	…	…	…	…	…
49	28	5	0	815	10.86	20.28	5	2	87	86	0.428	1.561
	28	5	0	823	10.73	19.17	3	0	89	84	0.496	1.672
	…	…	…	…	…	…	…	…	…	…	…	…
	28	5	0	813	10.89	18.89	5	2	83	87	0.379	1.398
50	42	16	1	707	12.81	22.5	10	7	93	78	0.752	3.287
	42	16	1	711	12.75	22.5	11	6	99	74	0.809	3.012
	…	…	…	…	…	…	…	…	…	…	…	…
	42	16	1	705	12.85	23.05	8	9	97	80	0.801	2.798

^1^ 0 for female and 1 for male.

**Table 7:** Preliminary judgment result of driving propensity.

Type of Driving Propensity	Number of Driver
Aggressive	03,08,12,13,20,23,27,30,33,35,41,44,46,47,50
Normal	02,06,07,09,11,15,16,17,22,25,28,29,34,37,38,40,43,48
Conservative	01,04,05,10,14,18,19,21,24,26,31,32,36,39,42,45,49

**Table 8:** Interpretation of total variance of each principal component.

Component	Initial Eigenvalues			Extracted Loading Sum of Squares		
	Total	Percentage of Variance	Cumulative Percentage	Total	Percentage of Variance	Cumulative Percentage
1	6.640	55.333%	55.333%	6.640	55.333%	55.333%
2	1.964	16.367%	71.700%	1.964	16.367%	71.700%
3	0.922	7.683%	79.382%	0.922	7.683%	79.382%
4	0.594	4.947%	84.329%	0.594	4.497%	84.329%
5	0.478	3.983%	88.311%	0.478	3.983%	88.311%
6	0.439	3.656%	91.967%			
7	0.309	2.574%	94.541%			
8	0.267	2.225%	96.766%			
9	0.181	1.506%	98.272%			
10	0.130	1.080%	99.352%			
11	0.073	0.610%	99.962%			
12	0.005	0.038%	100.000%			

**Table 9:** Score of each principal component.

Test Sample	First Principal Component	Second Principal Component	Third Principal Component	Fourth Principal Component	Fifth Principal Component
1	1.5103	−1.3385	0.9640	−3.3418	−0.7096
2	1.1362	−1.3688	0.8263	1.6209	0.0544
3	1.6318	−1.3398	1.0407	0.1206	0.4822
4	1.4625	−1.3542	0.9370	0.5988	0.8418
5	1.6846	−1.2961	0.8146	1.0791	0.2296
…	…	…	…	…	…
1001	−1.1198	1.1781	0.2660	−1.0783	−0.3280
1002	−1.3763	−0.0009	0.6690	−0.0936	0.1397
1003	−0.8410	0.0877	0.5144	0.8409	−0.0655
1004	−1.2461	0.0367	0.6016	−0.3086	−0.3139
1005	−1.0938	0.161	0.6866	−0.1223	1.3106
…	…	…	…	…	…
1996	−1.0287	−0.1602	0.8467	−0.7797	0.7990
1997	−0.9700	−0.1123	−0.6619	−0.8127	−1.0078
1998	−0.9648	−0.1336	0.6619	−0.8127	−1.0078
1999	−1.3439	−0.1849	0.8605	−0.5054	0.1205
2000	−1.5796	−0.2254	0.8340	−0.9133	−0.3929

**Table 10:** Input results for aggressive test samples.

Number	Output	Number	Output	Number	Output	Number	Output	Number	Output
1	0.0132	17	0.0072	33	0.0000	49	0.1339	65	1.0283
2	0.0085	18	0.0007	34	0.2195	50	0.0092	66	0.0000
3	0.0000	19	0.0000	35	0.1078	51	0.0000	67	0.0000
4	0.0000	20	0.0078	36	0.0000	52	0.1982	68	0.0000
5	0.0268	21	0.0000	37	0.0000	53	0.0000	69	0.1392
6	0.0091	22	0.0000	38	1.9938	54	0.0000	70	0.0000
7	0.9932	23	0.0000	39	0.2193	55	0.2012	71	0.0062
8	0.0000	24	0.0012	40	0.0016	56	0.0062	72	0.0301
9	0.0073	25	0.0035	41	0.0000	57	0.0032	73	0.0000
10	0.0000	26	0.0000	42	0.0021	58	0.0000	74	0.0081
11	0.1026	27	0.1067	43	0.0039	59	0.0000	75	0.0000
12	0.0000	28	0.0143	44	0.0093	60	0.0089	76	0.1061
13	0.0023	29	0.0000	45	0.2792	61	0.0002	77	0.0102
14	0.0000	30	0.0000	46	0.0000	62	0.1026	78	0.0000
15	0.2004	31	0.0000	47	0.0000	63	0.0401	79	0.0000
16	0.0017	32	0.0072	48	0.0000	64	0.0088	80	0.0000

**Table 11:** The final verification results of 20 drivers in the real vehicle experiment.

Number	Accuracy	Driving Propensity	Number	Accuracy	Driving Propensity
12	95.1%	Aggressive	25	93.3%	Normal
34	92.9%	Normal	44	95.1%	Aggressive
09	93.3%	Normal	32	96.2%	Conservative
42	94.5%	Conservative	26	92.9%	Conservative
17	92.2%	Normal	27	94.5%	Aggressive
03	96.2%	Aggressive	40	92.9%	Normal
21	95.1%	Conservative	38	92.2%	Normal
28	92.9%	Normal	47	96.2%	Aggressive
36	94.5%	Conservative	04	95.1%	Conservative
19	95.1%	Conservative	15	92.9%	Normal

**Table 12:** GRNN driving propensity identification results.

Number	σ	Accuracy/%	Number	σ	Accuracy/%
1	50	83.3	6	0.8	87.1
2	15	84.6	7	0.5	86.3
3	10	86.7	8	0.1	89.2
4	5	85.4	8	0.1	89.2
5	1	87.5	10	0.01	87.9

**Table 13:** BPNN driving propensity identification results.

Number	lr	goal	Accuracy/%
1	0.01	0.1	88.3
2		0.01	90.8
3		0.001	87.5
4	0.05	0.1	88.7
5		0.01	89.6
6		0.001	86.7
7	0.1	0.1	88.3
8		0.01	91.3
9		0.001	89.6

**Table 14:** FOA-GRNN driving propensity identification results.

Identification Results	Aggressive (Pre-Judgment Result)	Normal (Pre-Judgment Result)	Conservative (Pre-Judgment Result)
Aggressive (real result)	76	3	1
Normal (real result)	4	72	4
Conservative (real result)	1	5	74

**Table 15:** Various evaluation indicators of the FOA-GRNN driving propensity identification model.

Evaluation Indicators	Accuracy (%)	Precision (%)	Recall (%)	F1-Score (%)
Aggressive	92.5	93.83	95	94.56
Normal	92.5	90	90	90
Conservative	92.5	93.67	92.5	93.08

**Table 16:** GRNN driving propensity identification results.

Identification Results	Aggressive (Pre-Judgment Result)	Normal (Pre-Judgment Result)	Conservative (Pre-Judgment Result)
Aggressive (real result)	72	6	2
Normal (real result)	6	66	8
Conservative (real result)	3	7	70

**Table 17:** Various evaluation indicators of the FOA-GRNN driving propensity identification model.

Evaluation Indicators	Accuracy (%)	Precision (%)	Recall (%)	F1-Score (%)
Aggressive	85.83	88.89	90	89.44
Normal	85.83	83.54	82.5	83.02
Conservative	85.83	87.5	87.5	87.5

**Table 18:** BPNN driving propensity identification results.

Identification Results	Aggressive (Pre-Judgment Result)	Normal (Pre-Judgment Result)	Conservative (Pre-Judgment Result)
Aggressive (real result)	74	5	1
Normal (real result)	5	69	6
Conservative (real result)	2	6	72

**Table 19:** Various evaluation indicators of the BPNN driving propensity identification model.

Evaluation Indicators	Accuracy (%)	Precision (%)	Recall (%)	F1-Score (%)
Aggressive	89.58	91.36	92.5	91.93
Normal	89.58	86.25	86.25	86.25
Conservative	89.58	91.14	90	90.57

## Data Availability

The data presented in this study are available on request from the corresponding author. The data are not publicly available due to privacy.
